# Ratios of monocytes and neutrophils to lymphocytes in the blood predict benefit of CDK4/6 inhibitor treatment in metastatic breast cancer

**DOI:** 10.1038/s41598-023-47874-3

**Published:** 2023-12-02

**Authors:** Stefanos Ioannis Moukas, Sabine Kasimir-Bauer, Mitra Tewes, Hans-Christian Kolberg, Oliver Hoffmann, Rainer Kimmig, Corinna Keup

**Affiliations:** 1 Department of Gynecology and Obstetrics, University Hospital Essen, Hufelandstrasse 55, 45147 Essen, Germany; 2 Department of Medical Oncology, University Hospital Essen, 45147 Essen, Germany; 3 Department of Palliative Medicine, West German Cancer Center, University Hospital Essen, 45147 Essen, Germany; 4 Department of Gynecology and Obstetrics, Marienhospital Bottrop, 46236 Bottrop, Germany

**Keywords:** Oncology, Cancer, Breast cancer, Biomarkers, Predictive markers, Cancer, Breast cancer

## Abstract

Biomarkers to identify metastatic breast cancer (mBC) patients resistant to CDK4/6 inhibition (CDK4/6i) are currently missing. We evaluated the usefulness of the monocyte-to-lymphocyte ratio (MLR), the neutrophil–to-lymphocyte ratio (NLR) and the platelet-to-lymphocyte ratio (PLR) as predictive markers for de novo resistance to CDK4/6i. Various blood cell counts and MLR, NLR, PLR were recorded before treatment initiation (baseline) and four weeks later from 97 mBC patients receiving endocrine therapy (ET) alone or in combination with CDK4/6i. Binary blood cell count/ratios (mean = cut-off) were related to outcome using Cox regression. High MLR (p = 0.001) and high NLR (p = 0.01) at baseline significantly correlated with a shorter progression-free survival (PFS) in the CDK4/6i cohort, independent of any other clinical parameter as determined by multivariate Cox regression. Both, high MLR (p = 0.008) and high NLR (p = 0.043) as well as a decrease in PLR after four weeks of CDK4/6i first line treatment (p = 0.01) indicated a shorter overall survival. Moreover, decreasing PLR (p = 0.043) and increasing mean corpuscular volume (MCV; p = 0.011) within the first cycle of CDK4/6i correlated with a shorter PFS and decreasing MLR (p = 0.039) within the first cycle of first-line CDK4/6i was also correlated with shorter PFS. In summary, easily assessable blood cell parameter were shown to have predictive, monitoring and prognostic value and thus, could, in future, be used for individualized CDK4/6i therapy management. Most importantly, the imbalance of NLR and MLR at baseline might serve as predictive marker for de novo resistance to CDK4/6i in mBC patients.

## Introduction

Deregulation of the cyclinD-CDK4/6-Rb signaling has been linked to endocrine resistance in hormone receptor-positive (HR +) breast cancer (BC)^1,2^. Hence, inhibition of this signaling pathway in combination with endocrine therapy (ET) has resulted in improved progression-free (PFS) as well as overall survival (OS) when compared to ET alone^3–6^. Thus, CDK4/6 inhibitors in combination with ET, have become the treatment of choice for patients with metastatic (m), HR + /HER2-negative (HR + /HER2-) BC^7–11^ without visceral crisis.

However, de novo or acquired therapy resistance represents a significant clinical challenge^12^. A diverse range of potential resistance mechanisms, mostly shown to be acquired -not de novo existent-, like mutations in RB1, amplifications in p16, CDK4 or CDK6, activation of CDK2, the PIK3CA pathway, the MAPK pathway or the FGFR pathway, PTEN loss, aberrant cyclin-E1 or E2 signaling, FAT1 loss or other Hippo pathway involvement have been figured out^12,13^. Nonetheless, predictive markers for therapy decision making, based on insights into de novo resistance mechanisms, are still missing.

There is evidence that not only the tumor characteristics but also the host inflammatory responses are important for tumor growth and cancer progression^14–16^. In this regard, pro-inflammatory blood cells including white blood cells (WBC), namely lymphocytes, monocytes, neutrophils as well as platelets and, even more relevant, the monocyte-to-lymphocyte ratio (MLR), the neutrophil-to-lymphocyte ratio (NLR) and the platelet-to-lymphocyte ratio (PLR) have been reported as prognostic factors in different cancers^17–20^. In BC, their association with a reduced treatment efficacy, pathological complete remission and survival in localized disease, in the neo-adjuvant setting before and/or after chemotherapy as well as in mBC have already been documented^21–27^. In this context, regulatory and cytotoxic T cells, expressing high levels of CDK6 and RB1, as well as hematopoeitic stem cells were shown to be effected by CDK4/6 inhibition^28–31^. While anti-tumorigenic cytotoxic T cells are activated by CDK4/6i^29^, CDK4/6i resulted in reduced proliferation, survival and differentiation in hematopoietic stem cells^30^, one reason for the pronounced number of patients with neutropenia, leukopenia, thrombocytopenia and anemia as adverse effect under therapy with CDK4/6i.

Since the analysis of the immunological status within the tumor tissue requires high quality material that often is not available in the metastatic setting and the method itself is complex in clinical routine, we here evaluated whether the pro-inflammatory peripheral blood parameters MLR, NLR and PLR as well as other blood cell counts can serve as prognostic and monitoring marker for therapy guidance in mBC patients under CDK4/6i treatment. In addition, based on an existing subgroup of mBC patients receiving endocrine therapy alone, we also elucidated their role as a predictive parameter.

## Results

### Clinical characteristics

**Figure 1:**
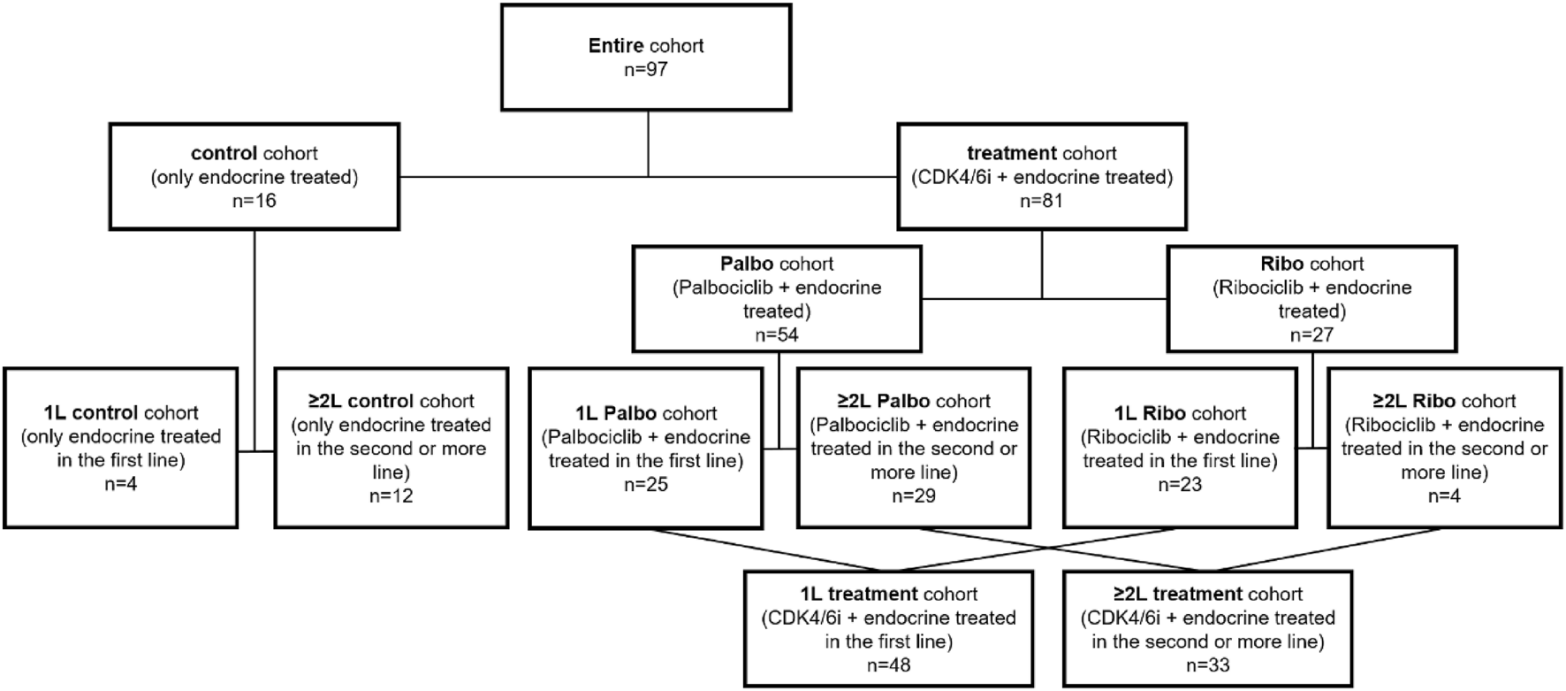
Scheme describing the sub-cohorts including the number of patients. 1L: first line; ≥ 2L: second or more line; Palbo:Palbociclib; Ribo:Ribociclib.

The entire cohort consisted of 97 patients, further detailed in Fig. 1, Table 1 and the method section ‘study population’. Within the CDK4/6i (treatment) cohort, patients received either Palbociclib (n = 54) or Ribociclib (n = 27). Patient cohorts were further stratified regarding the number of therapy lines before starting CDK4/6i and/or ET. In the CDK4/6i cohort, 48 patients received the treatment as first line therapy and 33 patients in second or more lines. The number of patients treated with Palbociclib in the first line (1L; n = 25) and Ribociclib in the first line (1L; n = 23; Fig. 1) were well balanced while patients treated in second or more lines (≥ 2L; n = 33) mostly received palbociclib (n = 29; Fig. 1).

**Table 1:** Patients characteristics.

Clinical characteristic	Total (%)
**Total number of patients**	97
**Age at time of metastasis diagnosis (years)**	
Mean (Min–Max)	60 (36–86)
**Follow-up time (baseline to last contact) (months)**	
Treatment cohort: Mean (Min–Max)	28 (2–70)
Control cohort: Mean (Min–Max)	31 (8–54)
**PFS (months)**	
Treatment cohort: Mean (Min–Max)	17 (1–57)
Control cohort: Mean (Min–Max)	16 (2–48)
**Clinical benefit (PFS > 6 months)**	
Yes	
Treatment cohort	63 (77.8)
Control cohort	9 (56.3)
No	
Treatment cohort	18 (22.2)
Control cohort	7 (43.8)
**Menopausal status**	
Premenopausal	16 (16.5)
Perimenopausal	15 (15.5)
Postmenopausal	63 (64.9)
Men	3 (3.1)
De novo **vs recurrent metastasis**	
De novo	45 (46.4)
Recurrent	52 (53.6)
**Disease-free interval (months) of the secondary metastatic patients**	
Mean (Min–Max)	116 (17–435)
**Number of sites of metastasis**	
1	41 (42.3)
2 or more	56 (57.7)
**Metastasis location**	
Non-visceral	52 (53.6)
Visceral	45 (46.4)
**Immunohistochemical subtype of metastasis**	
ER + , PR + , Her2-	41 (42.3)
ER + , PR-, Her2-	27 (27.8)
ER-, PR + , Her2-	1 (1.0)
HR + , Her2 +	2 (2.1)
Unknown	26 (26.8)
**Any therapy in metastatic setting before CDK 4/6i**	
< 2 prior therapy lines	75 (77.3)
> 2 prior therapy lines	22 (22.7)
**Analyzed treatment**	
CDK4/6i plus endocrine therapy	81 (78.6)
Ribociclib plus endocrine therapy	27 (27.8)
Palbociclib plus endocrine therapy	54 (55.7)
only endocrine therapy	16 (16.5)
**Endocrine therapy (in combination with CDK4/6i)**	
AI (Letrozol, Exemestan, Anastrozol)	50 (51.5)
SERD (Fulvestrant)	44 (45.4)
SERM (Tamoxifen)	3 (3.1)
**Therapy line in metastatic setting**	
First line	52 (53.6)
Second or more line	45 (46.4)

As detailed in Table 1, the mean age of the patients at the time of metastasis was 60 years (range 36–86 years). Most patients had a postmenopausal status (64.9%). 46.4% of patients had a de novo metastatic disease and 53.6% of patients had a recurrent metastatic disease, respectively. The latter group had a mean disease-free interval of 116 months (range 17–435 months). 42.3% of the patients had one site of metastasis compared to 57.7% of patients presenting with two or more metastatic sites with visceral metastasis documented for 46% and non-visceral metastasis in 53.6% of cases. The ER + PR + HER2- immunohistochemical subtype of the metastatic sites was the most frequently detected one while unknown in 26.8% of cases. The majority of patients (77.3%) received less than two therapy lines before the start of CDK4/6i and/or ET which consisted of aromatase inhibitors (51.5%), selective estrogen receptor degraders (45.4%) and Tamoxifen (3.1%), respectively (Table 1).

The mean follow-up time from baseline to last contact was 28 months (range 2–70 months) in the treatment and 31 months (range 8–54 months) in the control cohort. Mean PFS was 17 months (range 1–57 months) in the treatment and 16 months (range 2–48 months) in the control cohort. Clinical benefit, defined as PFS ≥ 6 months was reached in 56.3% of patients in the control group and 77.8% in the treatment cohort (Table 1).

### Distribution of absolute blood cell counts

**Figure 2:**
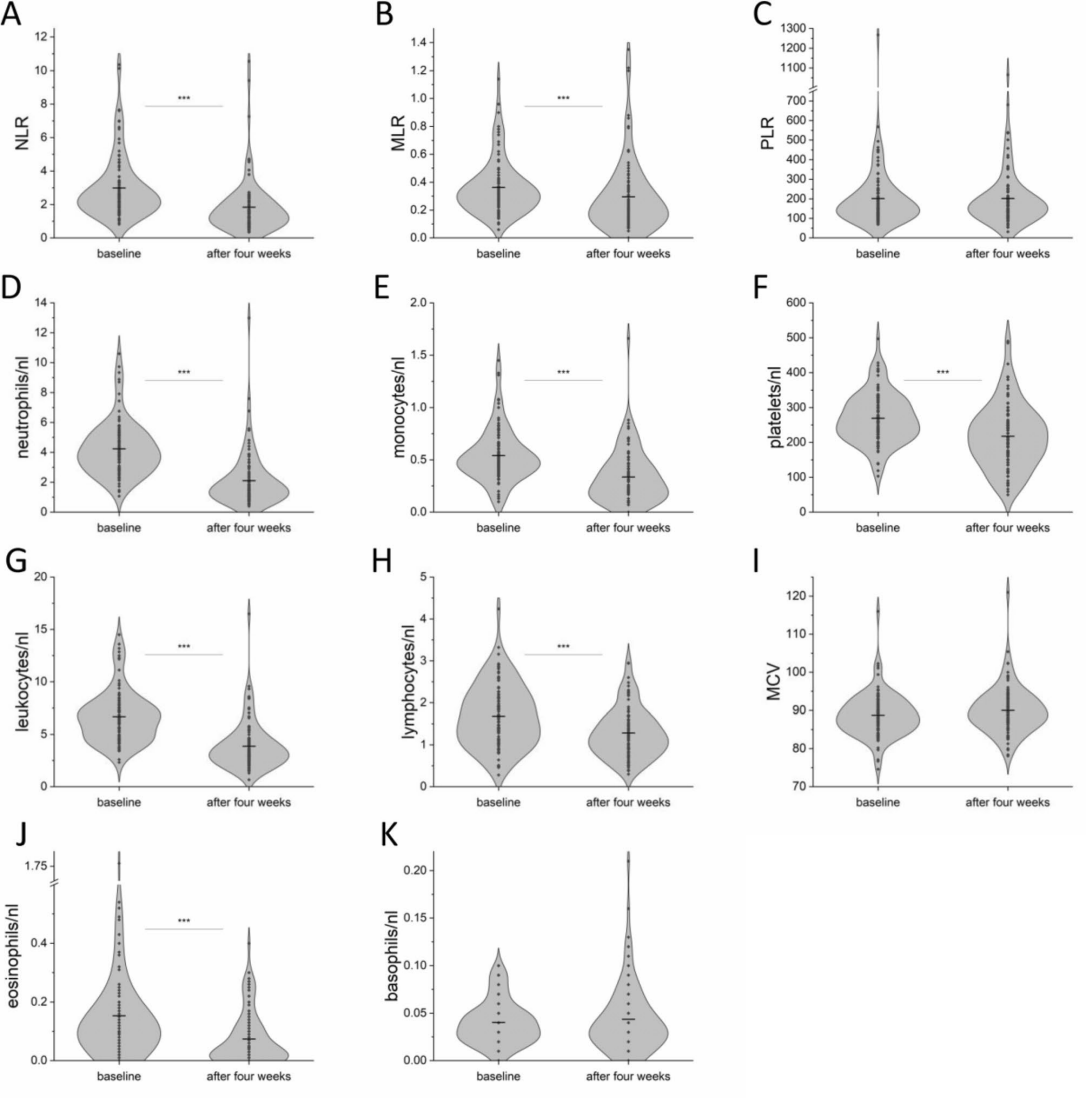
Distribution of absolute blood cell counts at baseline (n = 97) and after four weeks of therapy (n = 93). The violin plots show the distribution of blood cell counts and ratios of the entire cohort at baseline and after four weeks of therapy. The MCV (mean corpuscular volume) was reported in fl. The mean is marked as black line. Significantly different results regarding the distribution at baseline versus after four weeks were marked with asterisks (p < 0.001 = ***; by Wilcoxon signed-rank test). Descriptive statistics of all eleven blood parameters are listed in Supplementary Table 1.

The distribution of all MLR, NLR and PLR values as well as of all other blood cell counts and MCV from the entire cohort at both time points is depicted in Fig. 2, showing a significant decrease of neutrophils (p < 0.001), monocyctes (p < 0.001), platelets (p < 0.001), leukocytes (p < 0.001), lymphocytes (p < 0.001), eosinophils (p < 0.001) as well as MLR (p < 0.001) and NLR (p < 0.001), from baseline to four weeks under therapy by two-tailed Wilcoxon signed-rank test for matched samples (n = 93). The descriptive statistics of all eleven blood parameters at baseline in the entire cohort (mean, standard deviation, variance, minimum, maximum and skew) are listed in Supplementary Table 1—together with the healthy donor range according to Wakeman et al.^32^.

**Figure 3:**
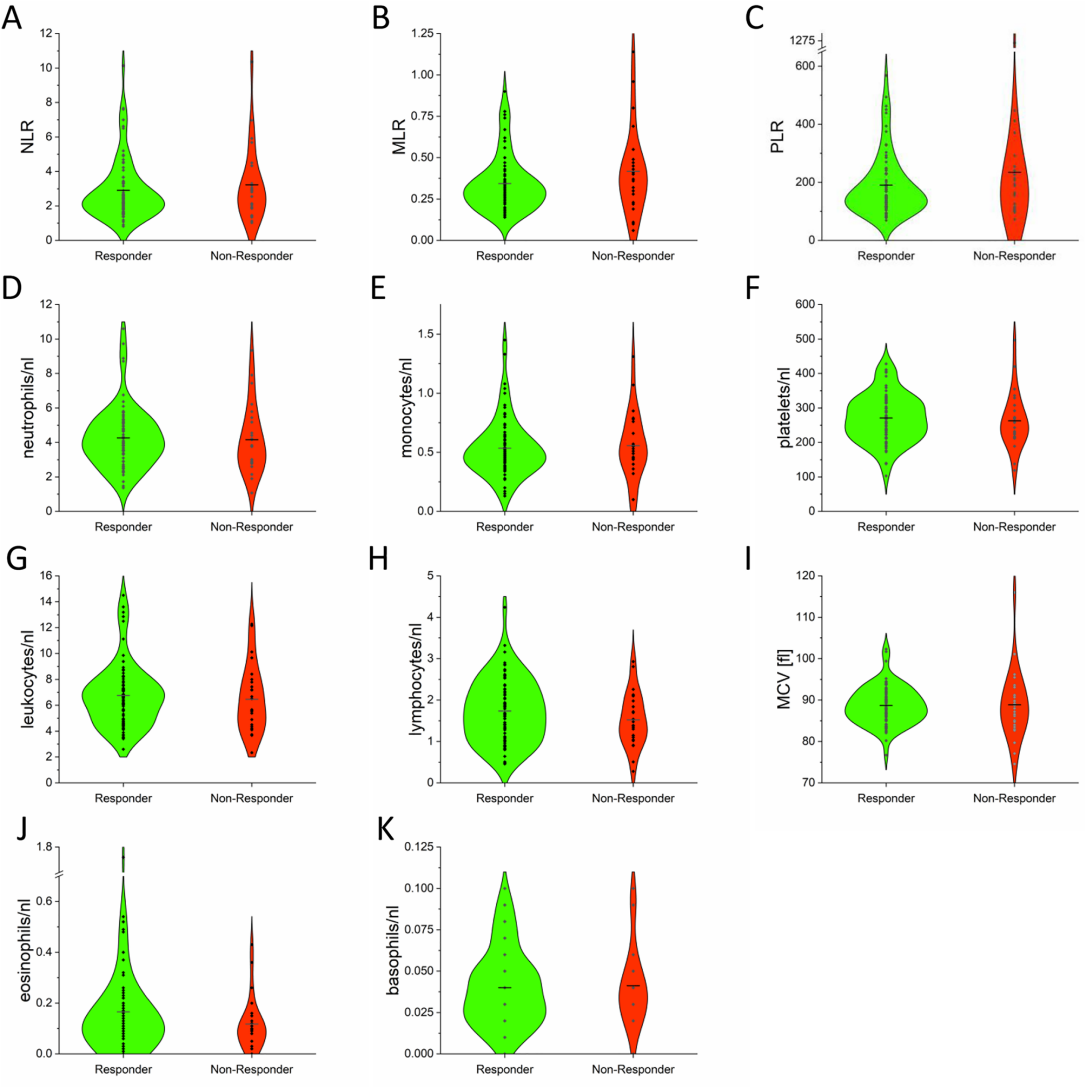
Distribution of absolute blood cell counts at baseline stratified into Responders (n = 72; green) and Non-Responders (n = 25; red). The mean is marked as black line. Associations of measurements regarding the distribution in Responders and Non-Responders were calculated by Mann–Whitney U test (no significant correlation detected).

No significant difference in the absolute blood cell counts, MCV or blood cell ratios at baseline was detected in Responders (n = 72) versus Non-Responders (n = 25) by two-tailed Mann–Whitney U test (Fig. 3).

**Figure 4:**
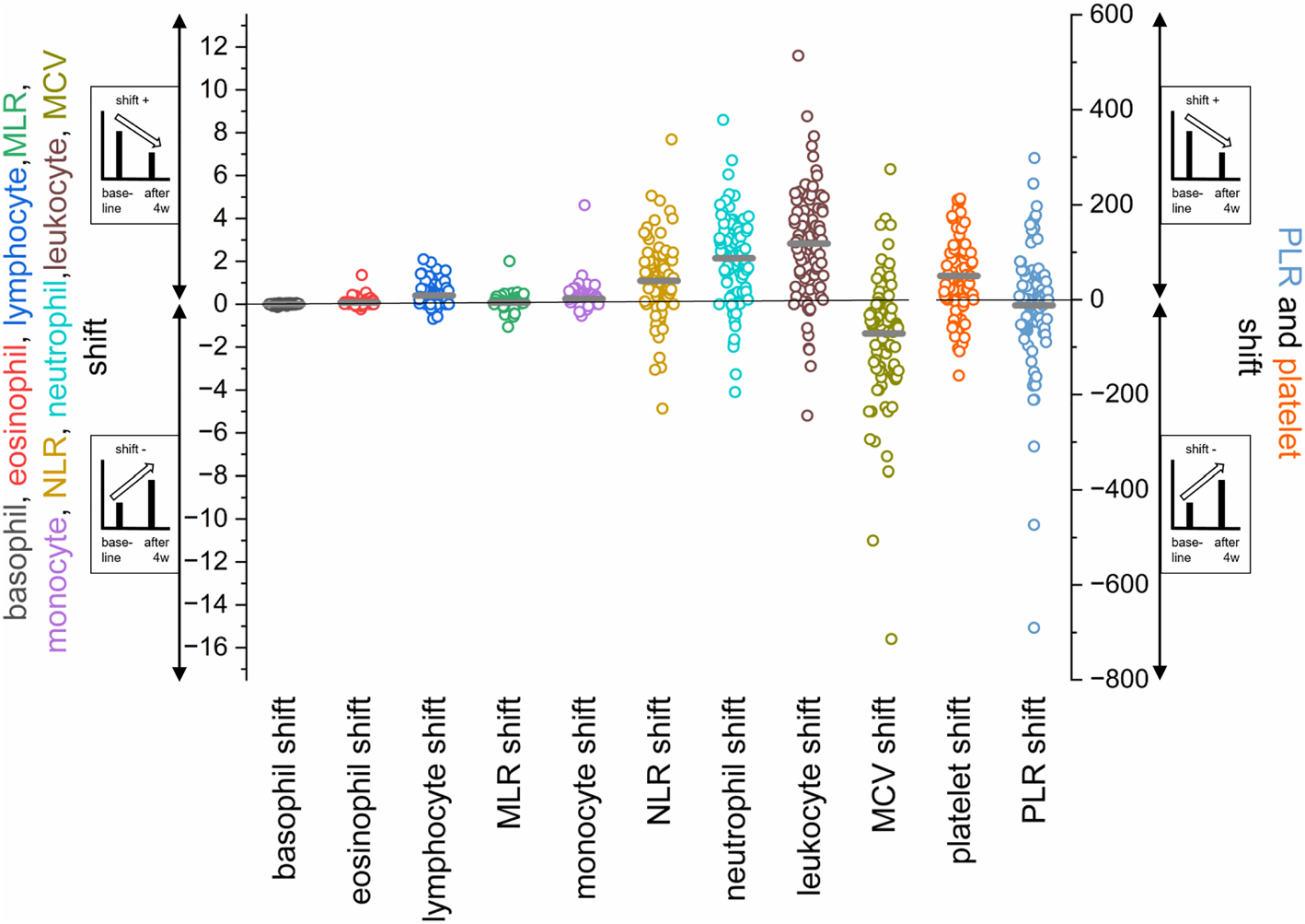
Distribution of the differences (shifts) in blood cell counts/ratios from baseline to four weeks under therapy (n = 93). Negative values (shift −) correspond to an increase of the respective measurements from baseline to four weeks of therapy. The mean is marked as grey line. Zero (no shift) is marked as black line over all categories. PLR and platelet shift values are linked to the right y-axis. All other values are linked to the left y-axis.

To state the dynamics of blood cell counts within a single patient under therapy, the differences of the respective measurements at baseline and after four weeks of therapy was calculated in matched samples (n = 93), here called shifts. As shown in Fig. 4, the dynamics of neutrophil and leukocyte number per nl under therapy were highly heterogeneous within the entire cohort with the majority of patients showing lower neutrophil and/or leukocyte numbers after four weeks of therapy. In contrast, the MCV was shown to be increasing under therapy in the majority of patients (Fig. 4).

### Correlation of blood cell counts/ratios with clinical parameters

When MLR-/NLR-/PLR-Low vs MLR-/NLR-/PLR-High groups at baseline (n = 97) were correlated with clinical characteristics (visceral metastasis, negative PR status, prior endocrine therapy, prior chemotherapy, de novo vs recurrent metastatic disease, one or more than one site of metastasis, age at therapy start) in the entire cohort, no significant differences were obtained by two-tailed Fisher’s exact test.

### MLR

#### MLR at baseline

**Figure 5:**
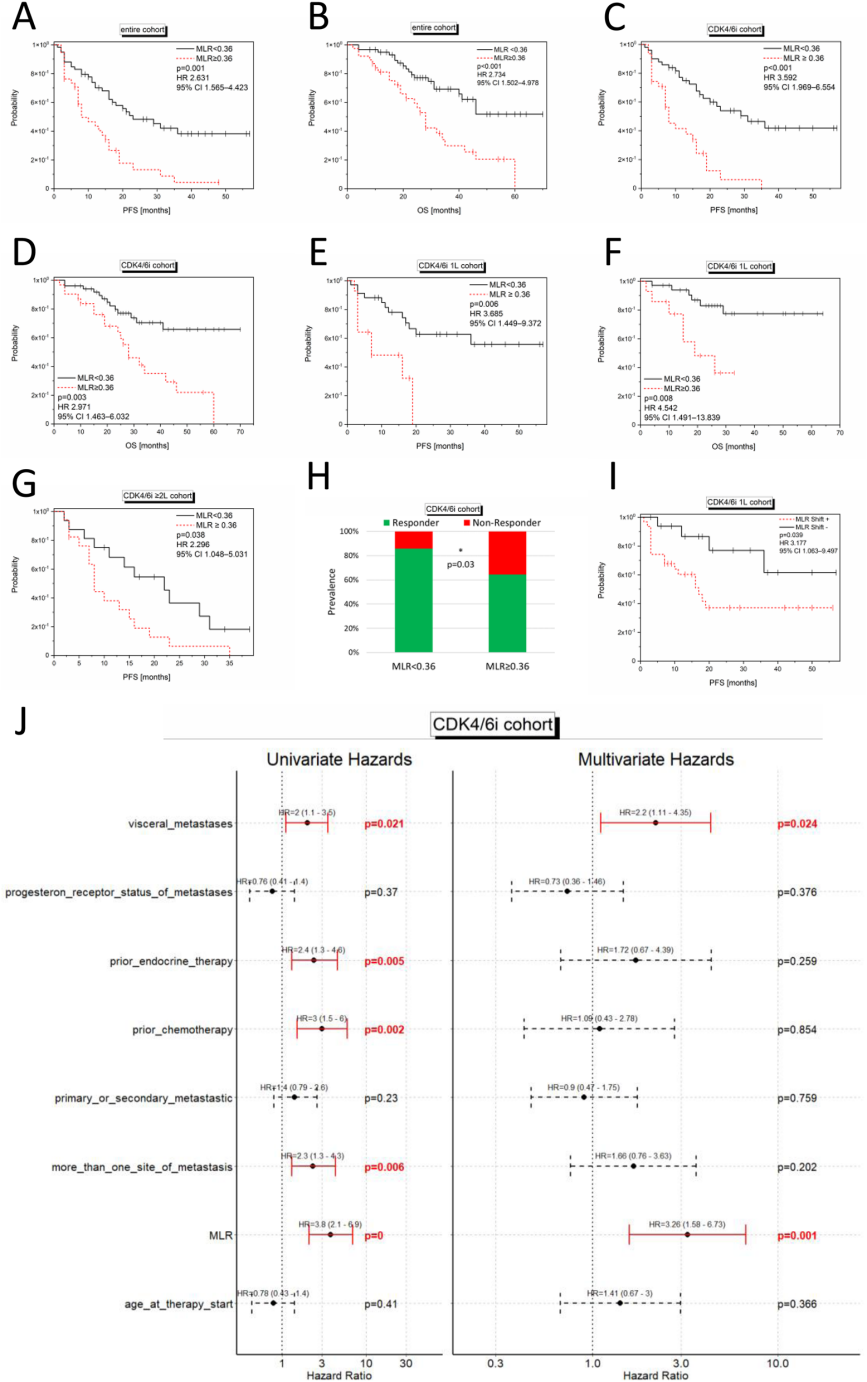
Significant correlations of MLR at baseline and MLR shift (from baseline to four weeks under therapy) with outcome. Kaplan Meier curves show PFS (**A, C, E, G, I**) and OS (**B, D, F**) in relation to the MLR (**A, B, C, D, E, F, G**, cut-off:0.36) and MLR shift values (**I**; cut-off:0) in the entire (**A, B**; n = 97), CDK4/6i (**C, D**; n = 81), CDK4/6i 1L (**E, F, I**; n = 48) and CDK4/6i ≥ 2L (**G**; n = 33) cohort. The p-values, HR and 95% CI values within the graphs show the results of the univariate Cox regression, while the log rank p-values are as follows: (**A**) p < 0.001; (**B**) p < 0.001; (**C**) p < 0.001; (**D**) p = 0.001; (**E**) p = 0.003; (**F**) p = 0.003; (**G**) p = 0.029; (**I**) p = 0.026. In (**H**), the significant difference between the prevalence of patients with MLR < 0.36 (n = 50) or MLR ≥ 0.36 (n = 31) regarding their clinical benefit of the CDK4/6i therapy is depicted (p = 0.03; exact Fishers’ test two-tailed). In (**J**), the multivariate Cox regression analysis is depicted showing a significant impact of MLR on PFS in the CDK4/6i cohort (n = 81).

In the entire cohort (n = 97), the mean value for MLR at baseline was 0.36 (range 0.06—1.14; n = 97; Fig. 2B). Using 0.36 as a cut-off, CDK4/6i treated patients (n = 81) with high MLR had a mean PFS of 11.59 months while patients with low MLR showed a mean PFS of 33.26 months. High MLR at baseline significantly correlated with a reduced PFS and OS in the entire cohort (log rank p < 0.001; univariate Cox regression: HR 2.631; 95% CI, 1.565–4.423; p = 0.001; and log-rank p < 0.001; univariate Cox regression: HR 2.734; 95% CI 1.502–4.978; p < 0.001; Fig. 5A and B), in the CDK4/6i cohort (log rank p < 0.001 univariate Cox regression: (HR 3.592; 95% CI 1.969–6.554; p < 0.001; and log rank p = 0.001; univariate Cox regression: HR 2.971; 95% CI 1.463–6.032; p = 0.003; Fig. 5C and D), in the CDK4/6i 1L cohort (log rank p = 0.003; univariate Cox regression: HR 3.685; 95% CI, 1.449–9.372; p = 0.006; and log rank p = 0.003; univariate Cox regression: HR 4.542; 95% CI 1.491–13.839; p = 0.008; Fig. 5E and F), as well as in the CDK4/6i ≥ 2L cohort (log rank p = 0.029; univariate Cox regression: HR 2.296; 95% CI, 1.048–5.031; p = 0.038; Fig. 5G) respectively.

CDK4/6i treated patients (n = 81) with high MLR at baseline were significantly more likely to achieve no clinical benefit (p = 0.03; exact Fishers’ test two-tailed; Fig. 5H) and multivariate Cox regression identified high MLR at baseline to be significantly associated with shorter PFS in the CDK4/6i cohort (HR 3.26; 95% CI 1.58–6.73; p = 0.001; Fig. 5J). In addition to MLR, the presence of visceral metastases was also an independent prognostic factor for PFS in the CDK4/6i cohort (multivariate Cox regression: HR 2.2; 95% CI 1.11–4.35; p = 0.024; Fig. 5J).

#### MLR shift

When MLR dynamics from baseline to four weeks of treatment were analyzed, decreasing MLR (MLR shift +) was significantly associated with a shorter PFS in the CDK4/6 1L cohort (n = 81; log-rank p = 0.026; univariate Cox regression: HR 3.177; 95% CI 1.063–9.3497; p = 0.039; Fig. 5I). No significant correlations were achieved with regard to OS in any cohort.

### NLR

#### NLR at baseline

**Figure 6:**
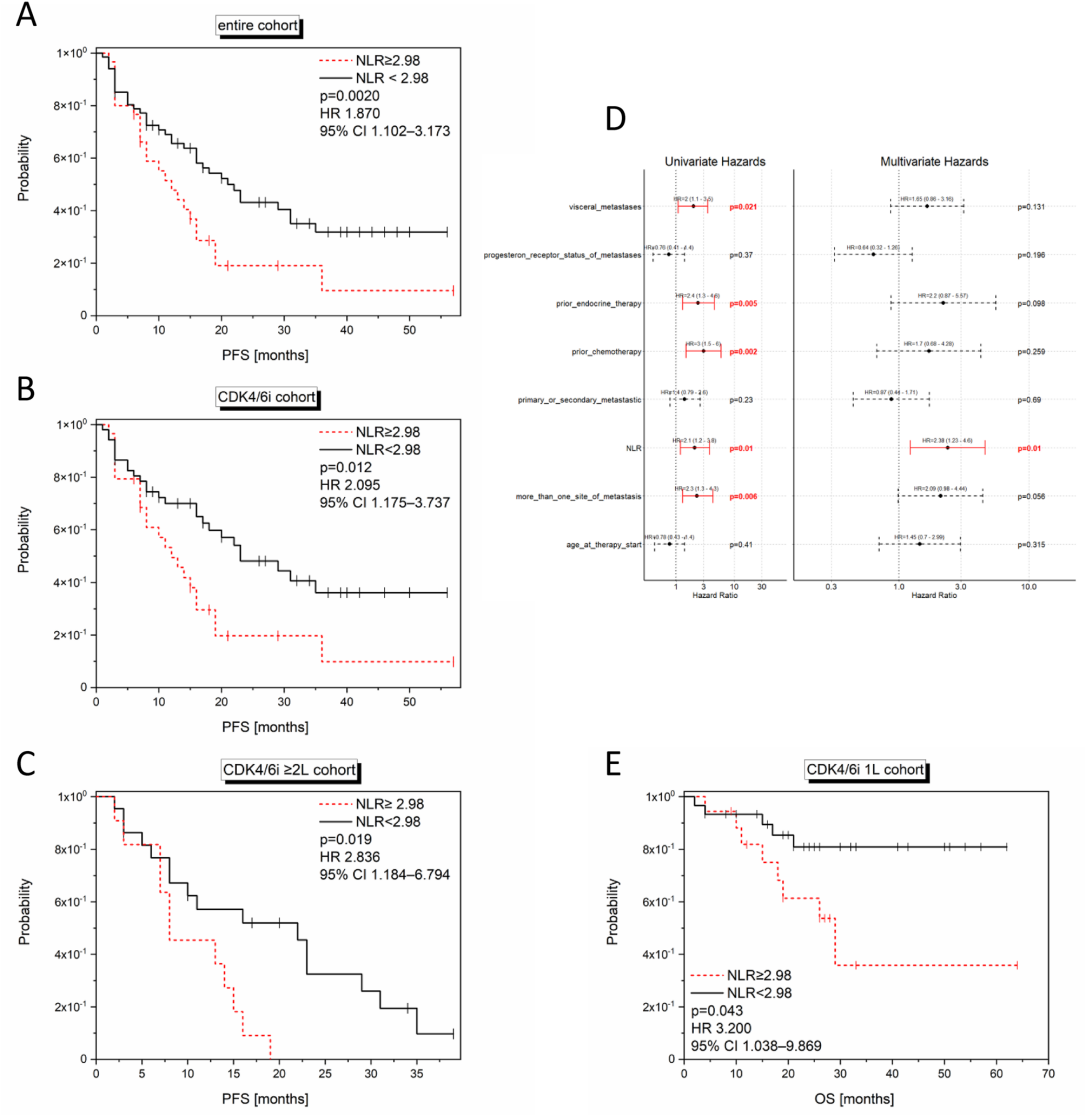
Significant correlations of the NLR at baseline with outcome. Kaplan Meier curves show the PFS (**A, B, C**) and OS (**E**) in relation to the NLR values (cut-off:2.98) in the entire (**A**; n = 97), CDK4/6i (**B**; n = 81), CDK4/6i 1L (**E**; n = 48) and CDK4/6i ≥ 2L (**C**; n = 33) cohort. The p-values, HR and 95% CI values within the graph show the results of the univariate Cox regression analysis, while the log rank p-values are as follows: (**A**) p = 0.016; (**B**) p = 0.009; (**C**) p = 0.019; (**E**) p = 0.032. In (**D**), the multivariate Cox regression analysis is depicted showing a significant impact of NLR on PFS in the CDK4/6i cohort (n = 81).

In the entire cohort (n = 97), the mean value for NLR at baseline was 2.98 (range 0.82–10.36; Fig. 2A). Using 2.98 as a cut-off, in the CDK4/6 cohort (n = 81), patients with high NLR at baseline had a mean PFS of 17.21 months while patients with low NLR achieved a mean PFS of 29.92 months. High NLR at baseline significantly correlated with a shorter PFS in the entire cohort (log-rank p = 0.016; univariate Cox regression: HR 1.870; 95% CI 1.102–3.173; p = 0.002; Fig. 6A), in the CDK4/6i cohort (log-rank p = 0.009; univariate Cox regression: HR 2.095; 95% CI 1.175–3.737; p = 0.012; Fig. 6B) and in the CDK4/6i ≥ 2L cohort (log-rank p = 0.012; univariate Cox regression: HR 2.836; 95% CI 1.184–6.794; p = 0.019; Fig. 6C). Multivariate Cox regression (HR 2.38; 95% CI 1.23–4.6; p = 0.01; Fig. 6D) confirmed NLR to be significantly associated with a shorter PFS in the CDK4/6i cohort (n = 81).

Reduced OS could be demonstrated for the CDK4/6i 1L cohort (n = 48), presenting with high NLR (log-rank p = 0.032; univariate Cox regression: HR 3.200; 95% CI 1.038–9.869; p = 0.043; Fig. 6E).

#### NLR shift

When NLR dynamics from baseline to four weeks of treatment were analyzed, no significant associations were found with regard to PFS.

### PLR

#### PLR at baseline

In the entire cohort (n = 97), the mean value for PLR at baseline was 201.0 (range: 29.31 – 1267.86; Fig. 2C). Using 201.0 as a cut-off, in the CDK4/6 cohort (n = 81), patients with high PLR at baseline had a mean PFS of 21.86 months while patients with low PLR achieved a mean PFS of 27.15 months. No significant results for PLR with regard to PFS were obtained for any cohort.

#### PLR shift

**Figure 7:**
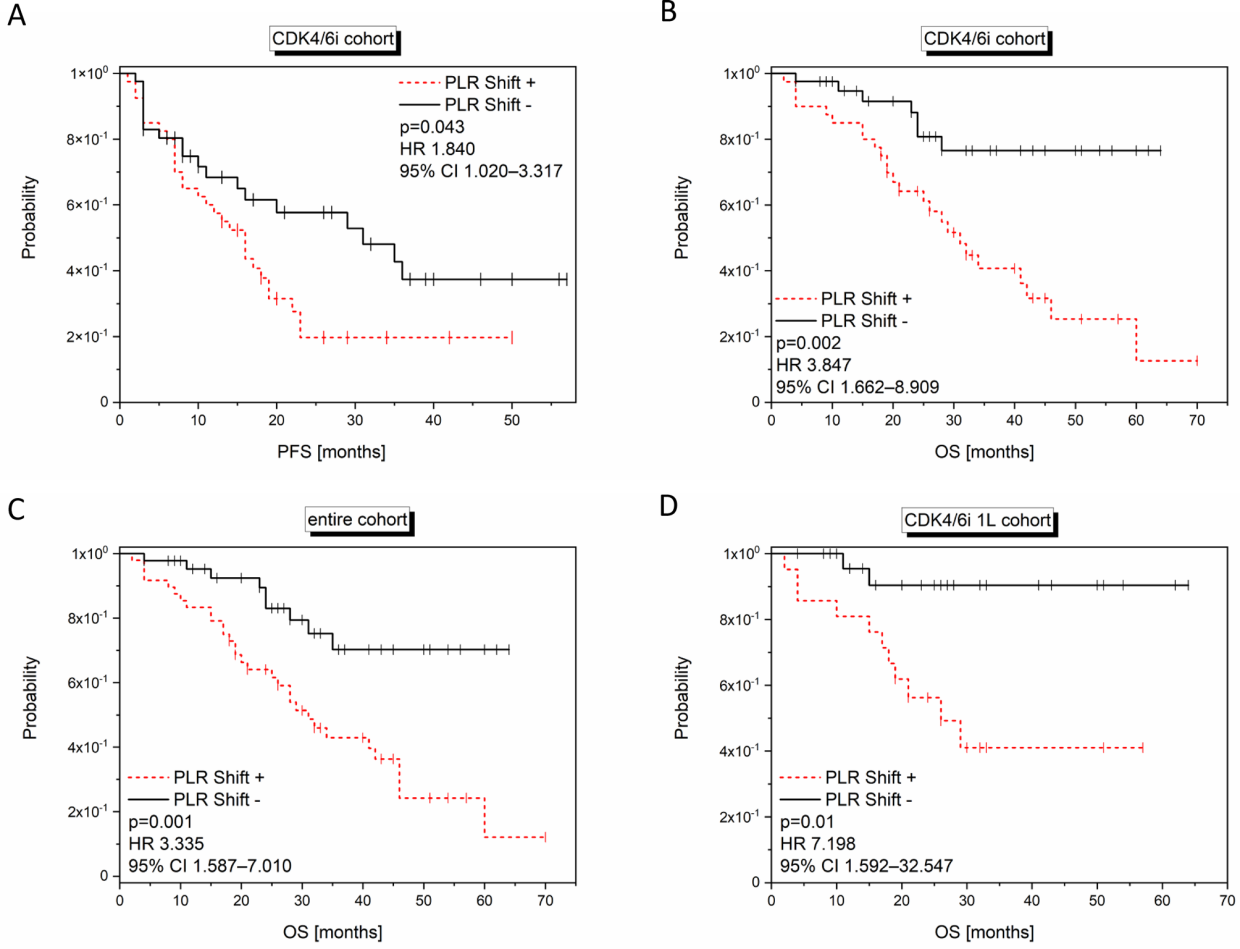
Significant correlations of the PLR shift with outcome. Kaplan Meier curves show PFS (**A**) and OS (**B**, **C**, **D**) in relation to the PLR shift values (cut-off:0) in the entire (**C**; n = 93), CDK4/6i (**A**, **B**; n = 81), CDK4/6i 1L (**D**; n = 48) cohort. The p-values, HR and 95% CI values within the graph show the results of the univariate Cox regression analysis, while the log rank p-values are as follows: (**A**) p = 0.036; (**B**) p < 0.001; (**C**) p = 0.001; (**D**) p = 0.003.

When PLR dynamics from baseline to four weeks of treatment were calculated, decreasing PLR (PLR shift +) was significantly correlated with a shorter PFS (log-rank p = 0.036; univariate Cox regression: HR 1.840; 95% CI 1.020–3.317; p = 0.043; Fig. 7A) and OS (log-rank p < 0.001; univariate Cox regression: HR 3.847; 95% CI 1.662–8.909; p = 0.002; Fig. 7B) in the CDK4/6i cohort (n = 81).

While PLR shift was not significantly related to OS in the control cohort (log-rank p = 0.883; data not shown; n = 12), decreasing PLR was identified in the entire cohort (log-rank p < 0.001; univariate Cox regression: HR 3.335; 95% CI 1.587–7.010; p = 0.001; Fig. 7C) and the CDK4/6i 1L cohort (log-rank p = 0.003; univariate Cox regression: HR 7.198; 95% CI 1.592–32.547; p = 0.010; Fig. 7D) to significantly correlate with shorter OS, besides the CDK4/6i cohort mentioned above.

### MCV

#### MCV at baseline

At baseline, the mean value for MCV was 88.8 (range: 74.6 – 116.0; Fig. 2I) in the entire cohort (n = 97). Using the mean value as cut-off, in the CDK4/6i cohort (n = 81) MCV high patients showed a mean PFS of 27.43 months and the MCV low patients of 23.13 months -meaning no significant PFS difference based on the MCV values at baseline.

#### MCV shift

**Figure 8:**
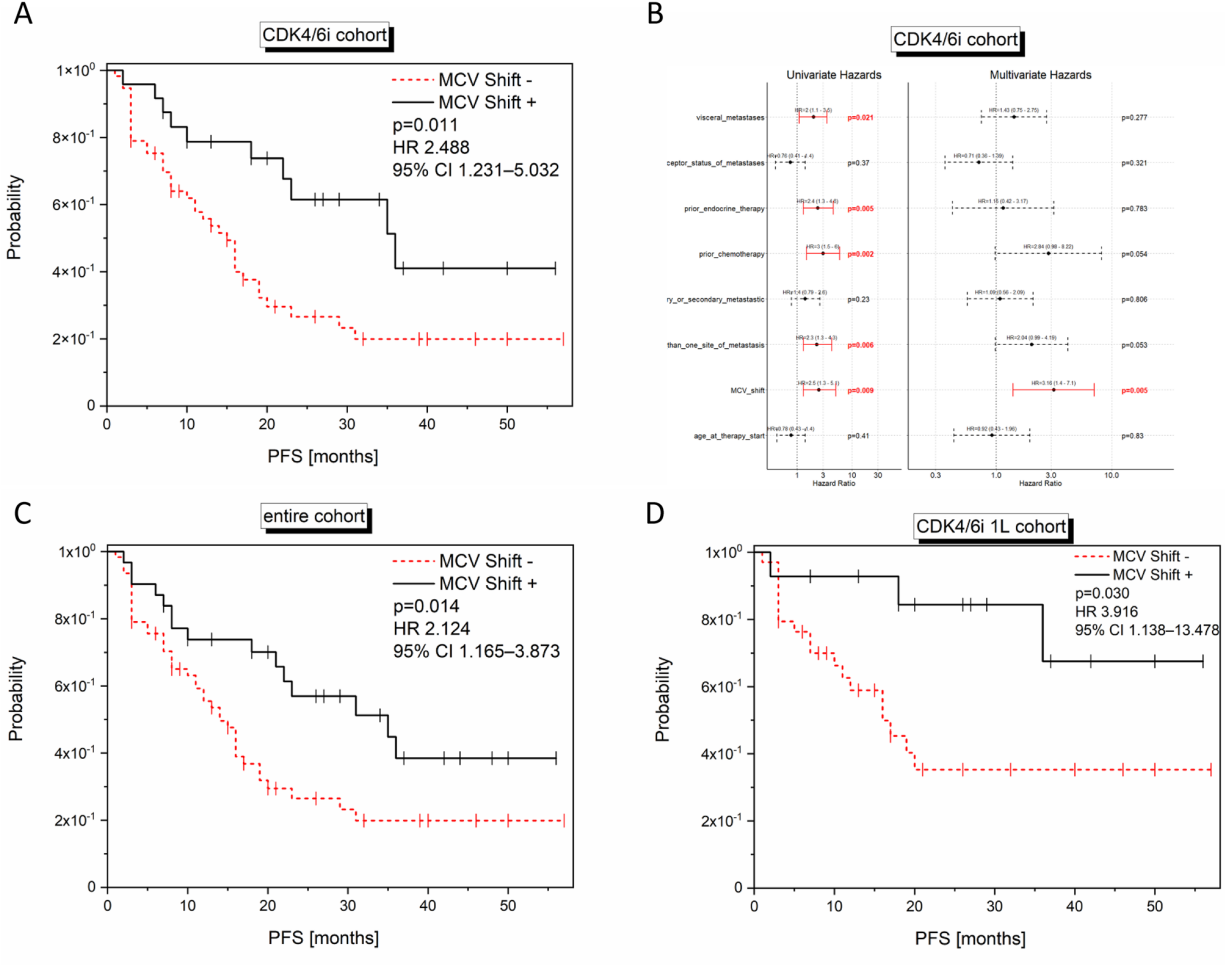
Significant correlations of the MCV shift with outcome. Kaplan Meier curves show PFS in relation to the MCV shift values (cut-off:0) in the entire (**C**; n = 93), CDK4/6i (**A**; n = 81), CDK4/6i 1L (**D**; n = 48) cohort. The p-values, HR and 95% CI values within the graph show the results of the univariate Cox regression analysis, while the log rank p-values are as follows: (**A**) p = 0.007; (**C**) p = 0.01; (**D**) p = 0.018. In (**B**), the multivariate Cox regression analysis is depicted showing a significant impact of MCV shift on PFS in the CDK4/6i cohort.

When MCV dynamics from baseline to four weeks of treatment were calculated, rising MCV values (MCV shift −) were significantly associated with a shorter PFS in the CDK4/6i cohort (n = 81; log-rank p = 0.007; univariate Cox regression: HR 2.488; 95% CI 1.231–5.032; p = 0.011; Fig. 8A), in the entire cohort (n = 97; log-rank p = 0.010; univariate Cox regression: HR 2.121; 95% CI 1.165–3.873; p = 0.014; Fig. 8C), and in the CDK4/6i 1L cohort (n = 48; log-rank p = 0.018; univariate Cox regression: HR 3.916; 95% CI 1.138–13.478; p = 0.030; Fig. 8D).

Multivariate Cox regression analysis only revealed increasing MCV within the first four weeks to be significantly associated with reduced PFS in the CDK4/6i cohort (n = 81; HR 3.16; 95% CI 1.4–7.1; p = 0.005; Fig. 8B).

## Discussion

In this retrospective analysis of blood cell counts and ratios, as easily detectable pro-inflammatory parameters, MLR and NLR at baseline were shown as independent predictive marker usable for therapy guidance in mBC patients to receive CDK4/6i plus ET. CDK4/6i treated patients with high MLR at baseline were significantly more likely to achieve no clinical benefit, indicating progressive disease within the first six months of therapy. Moreover, the prognostic value of MLR and NLR at baseline with regard to having a shorter OS was shown for patients receiving CDK4/6i as first line therapy after diagnosis of metastasis.

By comparison of matched samples at baseline and after four weeks of therapy, a significant decrease of neutrophils, monocyctes, platelets, leukocytes, lymphocytes, eosinophils as well as MLR and NLR under CDK4/6i was detected. In contrast, MCV increased in the majority of patients under therapy.

While decreasing PLR and increasing MCV within the first cycle of CDK4/6i correlated with shorter PFS, decreasing MLR within the first cycle was only correlated with a shorter PFS in the 1L CDK4/6i treated patients. These early-on-treatment assessments could be used as monitoring marker for therapy success.

Standard-of-care first-line therapy for patients with HR + /HER2- mBC without visceral crisis is CDK4/6i combined with ET, resulting in substantial PFS and OS benefits as well as maintained quality of life compared to ET alone^7,8^. HR + /HER2- mBC patients with visceral crisis receive chemotherapy as standard-of-care^7^. However, it is to question whether some patients without visceral crisis would benefit from chemotherapy more than from CDK4/6i plus ET. Although a meta-analysis revealed that no chemotherapy regimen showed increased PFS compared to CDK4/6i plus ET^33^, the Pearl study did not show superiority in PFS of Palbociclib + Fulvestrant vs. Xeloda^34^. Presented as an abstract, the Right Choice study further postulated that first-line Ribociclib plus ET increased PFS from 12.3 months to 24.0 months compared to chemotherapy in a patient cohort that included more than 50% of patients with visceral crisis^35^. These two studies question the standard-of-care and highlight the importance of individual factors that mediate the outcome under CDK4/6i.

At the moment, research focusses on these kind of individual factors as predictive markers to indicate de novo resistance to CDK4/6i therapy^36^. Predictive markers would enable individualized therapy approaches towards longer PFS, spared side effects and increased quality of life.

One of the factors that might influence the outcome of CDK4/6i is the composition of the tumor microenvironment^37^, because CDK4/6i was shown to effect the immune system. CDK4/6i triggers anti-tumor immunity by different ways. CDK4/6i activates tumor cell expression of endogenous retroviral elements, thus, increasing intracellular levels of double-stranded RNA. This in turn stimulates production of type III interferons and enhances tumor antigen presentation^28^. Enhanced tumor antigen presentation leads to increased anti-tumor immunity due to detection and killing of tumor cells by cytotoxic T cells. Second, the cytotoxic T cells themselves are also effected by CDK6 inhibition as it leads to de-repression of NFAT family transcription factors and consequently, increased cytotoxic T cell recruitment and enhanced T cell activation^29^. Both effects lead to cytotoxic T cell-mediated clearance of tumor cells. Third, CDK4/6 inhibitors markedly suppress the proliferation of regulatory T cells (Tregs) by reduced activity of the E2F target, DNA methyltransferase^28^. Consequently, the number of pro-tumorigenic Tregs decrease. In summary, CDK4/6 inhibitors decrease Treg proliferation but increase tumor infiltration and activation of cyctotoxic T cells leading to an overall enhanced anti-tumor immunity. Fourth, CDK4/6i also leads to reduced stem cell and progenitor cell proliferation mediated by reduced Notch signaling^30^. A consequence of these CDK4/6i effects might be the long-term reduction in different blood cell populations and the common adverse events like neutropenia and leukopenia.

With the knowledge of the numerous effects of CDK4/6i therapy on tumor cells, Tregs, cytotoxic T cells, and even stem and progenitor cells, it is reasonable to assume that the pretreatment status of the tumor immunity may have predictive value. NLR, MLR and PLR are pro-inflammatory signatures representing peripheral blood surrogates of the tumor immunity.

Comparison of the descriptive statistics of all blood parameters at baseline in the entire HR + /HER2- mBC cohort to the healthy donor reference ranges according to Wakeman et al.^32^ showed the minimal and maximal values of the entire HR + /HER2- mBC cohort within the healthy reference range for lymphocyctes, eosinophils and basophils. In contrast, in the entire HR + /HER2- mBC cohort, minimal and/or maximal values for neutrophils, monocyctes, platelets, leukocytes and MCV were outside the healthy reference range. The mean values of all mentioned eight blood parameters of the entire HR + /HER2- mBC cohort at baseline were within the healthy reference range.

### MLR data

We identified high MLR at baseline as independent predictive factor for reduced PFS in the CDK4/6i cohort and decreasing MLR within the first four weeks under therapy was associated with poor PFS in the CDK4/6i 1L cohort. To our knowledge, MLR has not been examined as predictive marker for CDK4/6i by any other group before. It is further to notice that we used a similar cut-off as other groups that studied MLR in mBC patients including all BC subtypes (0.36 in this project; 0.34^38^ and 0.28^39^).

In a mouse model representing lung metastases in the BC setting, it was shown that circulating monocyctes were reduced in number under CDK4/6i, but an increase in monocyte invasion was detected^40^. The decrease in circulating monocyte number was detected in CDK4/6i patients in our project as well.

### NLR data

A meta-analysis revealed that the NLR cut-off values in 15 analyzed studies ranged from 1.9 to 5.0^41^. The mean NLR value of 2.98, used in our entire cohort as cut-off, is in line with the majority of other studies that used 3.0 as cut-off^41^, further justified in one study as the optimal value to differentiate mBC patients with OS less or greater than 24 months^38^.

We identified high NLR at baseline as an independent predictive marker for shorter PFS in the CDK4/6i cohort. These results confirm the results shown recently as conference abstract, where high NLR at baseline was independently associated with lower PFS in 308 HR + /HER2- advanced BC patients receiving CDK4/6i therapy^42^. Similar results were demonstrated in a more stringent cohort of 126 1L CDK4/6i patients with a NLR cut-off of 2.53^43^. In a smaller cohort of 89 1L CDK4/6 treated HR + /HER2- mBC patients, high NLR (here defined > 3.7) was not found to be significantly correlated with worse PFS in a meeting abstract^44^. The latter two studies highlight the importance of evaluating markers in a large stringent cohort and only the use of consistent cut-off values will lead to reproducible results transferable into clinical practise. However, the evidence of high NLR at baseline as predictive marker for PFS under CDK4/6i therapy accumulates and might, thus, be usable to identify patients with de novo resistance to CDK4/6i due to the pretreatment status of the tumor immunity detected by blood surrogates.

In our study, we included a control cohort only receiving ET and found no significant lower PFS in patients with high NLR at baseline. However, in the entire cohort, including CDK4/6i treated and only endocrine treated patients, univariate Cox regression showed a significant association of high NLR with worse PFS, questioning the specificity of NLR with regard to the given therapy as predictive marker for CDK4/6i. Similarly, the results of two studies analyzing eribulin treated mBC patients also suggested high NLR to be a predictive marker for shorter PFS in mBC – receiving other treatment regimens than CDK4/6i^22,45^.

In this regard, it is to mention that clinical parameters influencing PFS should be integrated in the identification of a predictive marker, as done in this study by multivariate Cox regression analysis with clinical parameters also used in other CDK4/6i studies^46,47^. This is important because in a cohort of 263 mBC patients including all BC subtypes, a high NLR (defined as > 2.32) was significantly associated with worse PFS in univariate Cox regression but not in multivariate Cox regression analysis^48^.

Interestingly, Kim et al. determined NLR after one treatment cycle with high NLR to predict a reduced PFS^49,50^. However, this strategy is not usable for therapy decision making, because a predictive marker has to be evaluated before therapy start.

In addition to the predictive value of NLR, we and others^43^ showed a significant correlation of high NLR at baseline with shorter OS in the 1L CDK4/6i cohort, demonstrating the prognostic value of NLR.

### PLR data

Decreasing PLR from baseline to four weeks of therapy correlated with a shorter PFS and OS in the CDK4/6i cohort identifying PLR dynamics as a potential monitoring and prognostic marker.

PLR, not PLR dynamics, correlated with worse OS in a huge cohort of 2374 BC patients, using a cut off value > 300^51^. However, this group found high PLR not related to OS when analyzing only the luminal BC patients, which is in line with our results, since baseline PLR values in our luminal mBC patients before CDK4/6i showed no prognostic value.

Zattarin et al., described in a conference abstract that PLR at baseline and also after the first three treatment cycles related to worse PFS in 308 HR + /HER2- advanced BC patients receiving CDK4/6i^42^ while Weiner et al., presented in a meeting abstract a significant association between PLR at baseline and PFS using univariate und multivariate analysis in a more stringent cohort of 89 1L CDK4/6i treated CDK4/6 patients^44^. Both groups revealed a predictive value of PLR at baseline before CDK4/6i in mBC which we cannot confirm in our study.

### MCV data

Using an identical mean MCV value of 89.0 fl at baseline as described before^52^, the rising MCV under CDK4/6i in our patients confirmed already published results^52,55,56^. In contrast to the results of the other workgroups, our results showed a prolonged PFS in patients with increasing MCV even in multivariate Cox regression analysis.

### Monitoring and prognostic value

Despite the search for a predictive marker assessable at baseline for therapy decision making, we evaluated the dynamics of the blood cell counts and ratio under the first CDK4/6i cycle. NLR, MLR as well as the number of neutrophils, monocyctes, platelets, leukocytes, lymphocytes and eosinophils decreased significantly from baseline to four weeks under therapy. These dynamics might be explained by the CDK4/6i induced cell-cycle arrest in hematopoietic cells^30^ but also by the increased recruitment of cyctotoxic T cells^29^ as well as the proliferation repression of regulatory T cells^28^ by CDK4/6i which we can only speculate but not proof in this study.

In addition to the monitoring value of MCV shift under CDK4/6i, we here present the monitoring value of the MLR shift from baseline to four weeks under therapy in the 1L CDK4/6 cohort, that might also be used to identify therapy success during treatment. Furthermore, we detected a prognostic value of baseline MLR, NLR and PLR in the 1L CDK4/6 cohort suitable to assess the outcome early and adjust therapy management and follow-up care.

### Advantages and limitations of the study

Since this was a retrospective study, time points of blood cell count analysis varied in a number of cases for baseline and four-week analysis. Consequently, especially the short-term effects of one therapy free week after CDK4/6i therapy at the end of each cycle could have influenced the blood results. Despite evaluating different blood cell counts and ratios, detailed analysis of other inflammatory markers, eg. C-reactive protein, procalcitonin or other acute-phase proteins and Lactate dehydrogenase should also be taken into account in future studies. Unfortunately, these protein quantities were not available for our patients. Acute or chronic inflammatory diseases and/or cortison, novalgin or non-steroidal anti-inflammatory drug etc. intake was not documented for the included patients. Further prospective studies using fresh blood at the given time points would also allow for the quantification of B cells and T cells or the CD4 + /CD8 + cell ratio.

Although limited in sample size and not randomized for different treatment regimens, one advantage of our study is a control group to identify CDK4/6i specificity within our findings. To the best of our knowledge, this is the first study which included a control group to clearly identify CDK4/6i therapy specific predictive markers. One further advantage of this study are clearly defined sampling time points independent of dose reduction and drug holiday, which only take place after more than one therapy cycle.

We included patients that received CDK4/6i in the first line as well as in second or more lines, thus, the results of the latter group have to be interpreted with caution due to unknown effects of prior therapies on blood cell counts. The majority of patients (29/33) in the ≥ 2L CDK4/6i received Palbociclib plus ET and only four patients in this cohort received Ribociclib plus ET, thus, results obtained for patients with ≥ 2L CDK4/6i may be related more to Palbociclib then Ribociclib. However, our subgroup stratification clearly showed significances not only in the 1L CDK4/6i cohort, but also in the CDK4/6i cohort, consisting of 1L and ≥ 2L CDK4/6i treated patients. Consequently, we here demonstrate a broader range of implications for the clinical setting as our results are not specific for the number of therapy lines applied before start of CDK4/6i therapy, but can be applied to the entire HR + /HER2- mBC population receiving CDK4/6i. As we differentiated the 1L CDK4/6i cohort from the ≥ 2L cohort for the statistical analysis, reported 1L cohort results are as stringent as in other studies that only analyzed 1L CDK4/6i patients. The subgroup stratification into 1L and ≥ 2L CDK4/6i recently gained relevance with the publication of the SONIA trial results that challenged the need of CDK4/6i in the 1L^57^.

## Conclusion

Our study supports the known prognostic value of NLR and here validated its predictive value for therapy decision in mBC patients receiving CDK4/6i. To the best of our knowledge, we are the first to show the predictive and prognostic value of MLR in CDK4/6i treated patients while dynamics from baseline to four weeks under therapy for MLR, PLR and MCV revealed monitoring value in (1L) CDK4/6i treated patients.

Due to the attainability of these biomarkers and their valuable cost-effectiveness, they are adaptable in routine clinical practice and, after validation in larger cohorts and prospective trials with a consensus of meaningful cut-offs, MLR and NLR might fill the current gap of urgently needed predictive markers for CDK4/6i in HR + /HER2- mBC.

## Patients and methods

### Ethics and inclusion statement

This retrospective study, adhering to the REMARK guidelines, was conducted in the Department of Gynecology and Obstetrics, in collaboration with the Department of Medical Oncology, both at the University Hospital Essen, Germany and with the Marienhospital Bottrop, Germany. Together they are forming the Breast Cancer Center Essen I associated with the West German Tumor Center at the University Hospital Essen, Germany. The ethics committee of the Medical Faculty of the University Hospital Essen, Germany approved this study (ethic vote 12–5265-BO). In accordance with the declaration of Helsinki, written informed consent was obtained from all participants and the study did not result in any stigmatization, incrimination, discrimination or otherwise personal risk to participants. This study did not involve any health, safety, security or other risk to researchers. All authors read and approved the final manuscript.

### Study population

In total, 97 patients who were diagnosed with HR + /HER2- mBC between Mai 2015 and August 2021 were included in our evaluation. Blood specimens were collected before therapy start and after four weeks of treatment.

The eligibility criteria were as follows: age ≥ 18 years, measurable mBC, predicted life expectancy ≥ 2 months, Eastern Cooperative Oncology Group (ECOG) scores for performance status of 0–2, no severe uncontrolled co-morbidities or medical conditions, no second malignancies. Patients with symptomatic lymphangitic lung metastases, bone marrow replacement with associated cytopenia, leptomeningeal carcinomatous, or significant liver metastases with associated liver dysfunction, defined as having visceral crisis were excluded from the study. Patients had either primary tumors with estrogen (ER)-and/or progesterone (PR)-receptor positivity or biopsied metastases with estrogen (ER)- and/or progesterone (PR)- receptor positivity. Two patients had *ERBB2* over-amplification in their metastases, biopsied before CDK4/6i start. They first received chemotherapy, ribociclib and dual anti-HER2 blockade, followed by ET, ribociclib and dual anti-HER2 blockade when chemotherapy was completed (in the context of the Detect V study). Patients were either de novo metastatic or recurrent metastatic. Prior neoadjuvant and adjuvant treatment, radiation and all kinds of surgical intervention were permitted.

Patients received a CDK4/6 inhibitor [palbociclib (n = 54) or ribociclib (n = 27)] plus ET (n = 81, treatment group) or ET only (n = 16, control group). Patients received this treatment either in first line (1L) or in second or more lines (≥ 2L). Consequently, patients treated with therapy other than CDK4/6 inhibitors and/or ET after diagnosis of metastases were included in this study in the ≥ 2L treated cohort. In the latter group, patients` last therapy (chemotherapy, ET or other) had taken place at least three weeks before inclusion into this study. Patients who discontinued therapy due to adverse effects were excluded. Patient characteristics are listed in Table 1 and the detailed study design is shown in Fig. 1.

### Stratification of patients

Disease assessment was done by chest and abdomen computerized tomography (CT)-scans, magnetic resonance imaging and/or skeletal scintigram. PFS was defined as the time from baseline to disease progression. Responders were defined as patients with a PFS longer than six months, while Non-Responders had a PFS ≤ six months. All Responders were defined to have achieved clinical benefit from therapy. OS was defined as the time from baseline to death from any cause.

### Evaluation of blood cell counts

Blood values before treatment (± 4 days) as well as after four weeks of treatment (± 6 days) were retrospectively recorded. Blood values from patients presenting in the University Hospital Essen were measured in the central laboratory using a Sysmex XN-1000 system, based on fluorescence flow cytometry, a cyanide-free hemoglobin method, with hydrodynamically focused impedance technology and cumulative pulse height summation. Blood values from patients presenting at the Marienhospital Bottrop were obtained via the C23653—DxH 900–2 analysis system, based on flow cytometric analysis in near-native state with five light-scatter angles. The values of neutrophils, monocytes, platelets, leukocytes, lymphocytes, eosinophils and basophils were reported in absolute number per nanolitre (nl). The MCV (mean corpuscular volume) was reported in femtoliter (fl). MLR, NLR and PLR were calculated by division of the absolute number per nl of monocytes, neutrophiles and platelets through the absolute number of lymphocytes per nl. To binarize the quantitative measurements at baseline, the mean value within the entire cohort (treatment and control cohort) was used as cut-off: 0.36 for MLR, 2.98 for NLR and, 201.0 for PLR, respectively. The measurements after four weeks were binarized by the mean value within the entire cohort at this time point. To report changes within blood values from therapy start to four weeks on therapy, the respective values or ratios measured after four weeks were subtracted from the baseline values and defined as ‘shift’. These quantitative ‘shift’ values were binarized with the cut-off 0, meaning to differentiate between blood values/ratios that increased under therapy (shift −)) and the blood values/ratios that decreased under therapy (shift +).

### Statistical analysis

Statistical analysis was performed using IBM SPSS Statistics Version 28.0.0.0. The metric parameters age, PFS and OS as well as all continuous independent variables were checked via Kolmogorov–Smirnov and shapiro–wilk test for normal distribution. Wilcoxon signed-rank test was used for non-normally distributed continuous variables (all blood cell counts/ratios) to validate the correlation with regard to the time point baseline vs four weeks under therapy in matched samples. The Mann–Whitney U test was used for non-normally distributed continuous variables (all blood cell counts/ratios) to check the correlation with the nominal parameter ‘clinical benefit’. Fisher’s exact test was applied to identify correlations between binary blood cell count values and the nominal dependent parameter ‘clinical benefit’. All analyses were two-tailed and exact. Associations with PFS and OS were analyzed using univariate and multivariate Cox proportional hazards regression models as well as the log‑rank test. The multivariate Cox regression model adjusted for visceral metastasis, PR status of metastases, prior endocrine therapy, prior chemotherapy, de novo vs recurrent metastatic disease, more than one site of metastasis and age at therapy start. P-values < 0.05 were considered to indicate a statistically significant difference in al tests (alpha level = 0.05). The bar chart was generated by Mircosoft Excel. Kaplan Meier curves and violin plots were generated by OriginPro 2022. The forest plots regarding uni- and multivariate hazard ratios were generated by R.

### Supplementary Information

Supplementary Information.

## Data Availability

The dataset generated and analysed during the current study is available in the Open Science Framework repository as .csv, .xlsx and .sav document, https://osf.io/p79yt, https://doi.org/10.17605/OSF.IO/P79YT.

## Abbreviations

≥ 2L: Second or more line
1L: First line
AI: Aromatase inhibitor
BC: Breast cancer
CDK4/6i: CDK4/6 inhibition
CI: Confidence interval
CT: Computed tomography
ER: Estrogen receptor
ET: Endocrine therapy
fl: Femtoliter
HER2-: Human epidermal growth factor receptor 2 negative
HR: Hazard ratio
HR +: Hormone receptor positive
m: Metastatic
mBC: Metastatic breast cancer
MCV: Mean corpuscular volume
MLR: Monocyte-to-lymphocyte ratio
nl: Nanoliter
NLR: Neutrophil–to-lymphocyte ratio
OS: Overall survival
Palbo: Palbociclib
PFS: Progression-free survival
PLR: Platelet-to-lymphocyte ratio
PR: Progesteron receptor
RB1: Retinoblastoma protein 1
Ribo: Ribociclib
SERD: Selective estrogen receptor degrader
SERM: Selective estrogen receptor modulator
WBC: White blood cell
