# Supplementary material for: Ratios of monocytes and neutrophils to lymphocytes in the blood predict benefit of CDK4/6 inhibitor treatment in metastatic breast cancer
Source: Sci Rep. 2023 Dec 2;13:21262. doi: 10.1038/s41598-023-47874-3 (PMC10692150; doi:10.1038/s41598-023-47874-3)
Supplement: Supplementary file 1 — Supplementary Information. [file 41598_2023_47874_MOESM1_ESM.pdf]

Manuscript: Ratios of monocytes and neutrophils to lymphocytes in the blood predict benefit of CDK4/6 inhibitor treatment in metastatic breast cancer
by Stefanos Moukas, Sabine Kasimir-Bauer, Mitra Tewes, Hans-Christian Kolberg, Oliver Hoffmann, Rainer Kimmig, Corinna Keup
Supplementary Table 1: Descriptive statistics of absolute blood cell counts at baseline in the entire cohort compared to the healthy range (abstracted from Wakeman et al., 2007).

		NLR	MLR	PLR	neutrophils [nl]	monocytes [nl]	platelets [nl]	leukocytes [nl]	lymphocytes [nl]	MCV	eosinophils [nl]	basophils [nl]
**entire HR+/HER2- mBC cohort at baseline**	mean	2,99	0,36	201,24	4,25	0,54	269,35	6,71	1,69	88,83	0,15	0,04
	standard deviation	1,86	0,20	151,82	1,82	0,26	72,45	2,50	0,74	5,68	0,20	0,02
	variance	3,47	0,04	23048,42	3,32	0,07	5249,08	6,23	0,55	32,22	0,04	0,00
	min	0,82	0,06	69,31	1,07	0,10	103,00	2,33	0,28	74,60	0,00	0,00
	max	10,36	1,14	1267,86	10,60	1,45	497,00	14,50	4,24	116,00	1,76	0,10
	skew	1,91	1,52	4,10	1,17	1,14	0,41	1,02	0,51	1,18	5,55	0,76
**healthy range** (according to Wakeman et al., 2007)	range min				1,70	0,20	180,00	3,60	1,00	85,00	0,00	0,00
	range max				6,20	0,80	380,00	9,20	3,40	98,00	0,40	0,10
